# Is web interviewing a good alternative to telephone interviewing? Findings from the International Tobacco Control (ITC) Netherlands Survey

**DOI:** 10.1186/1471-2458-10-351

**Published:** 2010-06-18

**Authors:** Gera E Nagelhout, Marc C Willemsen, Mary E Thompson, Geoffrey T Fong, Bas van den Putte, Hein de Vries

**Affiliations:** 1 Maastricht University/CAPHRI, PO Box 616, 6200 MD Maastricht, the Netherlands; 2 STIVORO for a smoke free future, PO Box 16070, 2500 BB The Hague, the Netherlands; 3 University of Waterloo, 200 University Avenue West, Waterloo, Canada; 4 Ontario Institute for Cancer Research, 101 College Street, Toronto, Canada; 5 University of Amsterdam, PO Box 19268, 1000 GG Amsterdam, the Netherlands

## Abstract

**Background:**

Web interviewing is becoming increasingly popular worldwide, because it has several advantages over telephone interviewing such as lower costs and shorter fieldwork periods. However, there are also concerns about data quality of web surveys. The aim of this study was to compare the International Tobacco Control (ITC) Netherlands web and telephone samples on demographic and smoking related variables to assess differences in data quality.

**Methods:**

Wave 1 of the ITC Netherlands Survey was completed by 1,668 web respondents and 404 telephone respondents of 18 years and older. The two surveys were conducted in parallel among adults who reported smoking at least monthly and had smoked at least 100 cigarettes over their lifetime.

**Results:**

Both the web and telephone survey had a cooperation rate of 78%. Web respondents with a fixed line telephone were significantly more often married, had a lower educational level, and were older than web respondents without a fixed line telephone. Telephone respondents with internet access were significantly more often married, had a higher educational level, and were younger than telephone respondents without internet. Web respondents were significantly less often married and lower educated than the Dutch population of smokers. Telephone respondents were significantly less often married and higher educated than the Dutch population of smokers. Web respondents used the "don't know" options more often than telephone respondents. Telephone respondents were somewhat more negative about smoking, had less intention to quit smoking, and had more self efficacy for quitting. The known association between educational level and self efficacy was present only in the web survey.

**Conclusions:**

Differences between the web and telephone sample were present, but the differences were small and not consistently favourable for either web or telephone interviewing. Our study findings suggested sometimes a better data quality in the web than in the telephone survey. Therefore, web interviewing can be a good alternative to telephone interviewing.

## Background

Web interviewing is becoming increasingly popular worldwide, because it has several advantages over telephone and paper-and-pencil interviewing. Web interviewing is less costly than telephone interviewing, and allows for lengthier questionnaires, a shorter fieldwork period, and the inclusion of visual stimuli [1-3]. In comparison with paper-and-pencil interviewing, web interviewing requires no data entry, presents one interview question at a time, and can use complicated skip patterns [4]. However, there are concerns that web surveys may yield data of lower quality. According to the 'total survey error' framework, data quality of surveys can be threatened by sampling error, coverage error, non-response error, and measurement error [5].

*Sampling error *occurs when a sample of the population is surveyed rather than the entire population [5]. Due to sampling error, point estimates of a sample study will not be precisely the same when another sample or the entire population is surveyed. When using probability samples, sampling error variability can be evaluated and described in terms of confidence intervals. However, web samples are not always created using probability sampling, but are often based on self selection. Self selection may result in a systematic bias, and the associated variability cannot reliably be described with a confidence interval.

*Coverage error *occurs when the list or frame from which the sample is taken does not correspond to the population of interest [5]. This error is for example present in a web survey when the findings are generalised to the entire population in a country were not everyone has internet access. Studies from the United States and Canada have shown that populations with internet access differed from populations without internet access, with those with internet access being younger, more often employed, less often part of a minority group, having more education, and having a higher income [6-10].

*Non-response error *occurs when not all sampled respondents participate in the survey [5]. When the actual respondents are not a random part of the initial sample, the respondents may not be representative of the population of interest. This was for example found in a U.S. web sample, where the responding part contained more females, non-minorities, higher educated and middle-aged respondents than the non-responding part [7].

*Measurement error *occurs when the measure employed is not an accurate or unbiased measure of what was to be measured, or is variable, lacking in precision or reliability [5]. The mode of data collection is an important factor here, because it determines the way in which questions are asked or presented [3]. Several studies have shown that web and telephone interviewing yield different results [2,11,12]. However, measurement error differences due to interviewing mode are mostly minor [6,13-15]. An example of measurement error differences due to interviewing mode is more socially desirable responding in telephone than in web surveys, which possibly happens due to the feeling of less anonymity with telephone than with web interviewing [2,12,16]. There are indications that telephone interviewing results in less reliable and valid measures than paper-and-pencil interviewing [13] and in more complicated factor structures than web interviewing [11].

This study focuses on differences in coverage error, non-response error, and measurement error of a national survey on smoking related behaviours, beliefs, and knowledge that was conducted in parallel by web and by telephone in the Netherlands. The Netherlands is a country where almost the whole population has internet access [17] and where web surveying is common practice. Although this correspondence between frame and population might be expected to lead to less survey error for web surveys in the Netherlands, this question has not so far been studied.

Although there have been few studies of survey mode effects carried out in the area of tobacco control, first reports indicate that smoking related questions can be reliably answered online [18-20]. There is evidence that smokers are less likely to have access to the internet and that those who have access use it less often than non-smokers [9,21]. However, it has also been found that smokers are more likely to prefer online research [22].

The current study is part of the International Tobacco Control Policy Evaluation (ITC) Project, which studies the effects of tobacco control policies on the attitudes and behaviours of smokers in 20 countries. The standard ITC survey mode in high-income countries is telephone, by means of random digit dialling. The ITC Netherlands Survey, which began in 2008, differs from other ITC surveys in that a mixed mode design was used. Some respondents were surveyed using telephone interviewing, but the majority of respondents were surveyed by web interviewing using samples drawn from a large population-based internet panel. The ITC Netherlands Survey will be used to assess differences in survey data quality on smoking when using web versus telephone interviewing.

Findings of the ITC Project are used to generalise to the population of smokers in different countries. Therefore, using a mode of interviewing that yields representative results is a very important part of the project. An important objective of tobacco control research in general is studying differences between smokers of different socioeconomic status groups. Smokers of lower socioeconomic status groups are less likely to quit smoking [23,24], as is evidenced by lower scores on determinants of smoking cessation [25]. In this study we will therefore test whether the differences between smokers of different socioeconomic status groups in determinants of smoking cessation are present in both the web and telephone survey.

We assessed differences in coverage error, non-response error and measurement error. The following research questions were addressed: (1) Were there more indications of coverage error in the web sample or in the telephone sample? (2) Were there more indications of non-response error in the web sample or in the telephone sample? (3) Were there indications of measurement error differences between the web sample and the telephone sample?

## Methods

The ITC Netherlands Survey was cleared for ethics by the Human Research Ethics Committee of the University of Waterloo and the Central Committee on Research Involving Human Subjects in the Netherlands.

### Web sample

The web respondents were drawn from TNS NIPObase, a large probability-based database with over 140,000 potential Dutch web respondents who have indicated their willingness to participate in research on a regular basis. TNS NIPObase panel members are actively recruited by TNS NIPO. People cannot apply for participation, which results in a low number of professional and inattentive respondents [26]. Web panel members are recruited by phone or mail, but not by internet. A screening procedure selected respondents that met the inclusion criteria for the ITC Netherlands Survey: being a monthly smoker of manufactured or roll-your-own cigarettes and having smoked at least 100 cigarettes in their lifetime. Quotas on gender, geographic region, household size, and education were determined from the 2007 Dutch Continuous Survey of Smoking Habits (DCSSH) to get a sample that was representative of Dutch smokers aged 15 years or older. The DCSSH is a national surveillance survey on smoking with weekly measurements of over 4,000 smoking respondents in 2007 and uses web respondents from TNS NIPObase. An announcement e-mail about the web survey was sent to 2,331 smoking respondents from TNS NIPObase. The announcement e-mail contained information about the subject of the survey, the time needed to fill it in, and the reimbursement that respondents could receive. It also contained a link to the website with conditions from TNS NIPObase. TNS NIPObase panel members had to read and agree to these conditions before becoming a panel member. The regulations state that members' personal information would be protected and that their participation in the surveys was voluntary. The web survey was completed from April 16 to April 25, 2008 by 1,820 respondents of 15 years and older. We analysed the data from 1,668 respondents who were 18 years and older. The respondents received compensation for their participation in the survey by earning points for every answered question, as is standard procedure in the TNS NIPObase web panel. The points could be exchanged into money, which ranged between 5 and 7 Euro for this survey.

### Telephone sample

The starting point of sampling the telephone respondents was the TNS NIPObase, which contains over 80,000 respondents with fixed line telephone numbers. This database is nationally representative on postal codes and matching telephone numbers. A sample was drawn by randomly taking telephone numbers from the database. The last two digits of each randomly drawn number were automatically replaced by two random digits. In total 28,563 numbers were called by the interviewers. When an interviewer reached an individual on the telephone, the interviewer asked how many people in the household fit the inclusion criteria: those reporting smoking at least monthly and who had smoked at least 100 cigarettes in their lifetime. In households where more than one person fit the criteria, the interviewer asked to speak with the person whose birthday was coming up next. The fieldwork took place from March 12 to April 26, 2008 and resulted in 404 completed interviews. The average telephone interview took 40 minutes. Each telephone respondent received a reimbursement of 10 euro by mail after completing the survey.

### Measurements

The ITC Netherlands web and telephone surveys consisted of 264 questions on demographics, smoking behaviour, and related issues, with extensive sections on tobacco control policies such as health warnings, smoke-free laws, advertising, and prices paid for cigarettes. Fong et al. [27] provides a description of objectives of the ITC Project Surveys, and Thompson et al. [28] provides a description of the methods used.

Basic demographic variables that have a known association with smoking cessation [23,24] were measured: gender, age, marital status, and educational level. Educational level was measured as a proxy of socioeconomic status and was categorized into three groups: low (primary education and lower pre-vocational secondary education), medium (middle pre-vocational secondary education and secondary vocational education), and high (senior general secondary education, (pre-)university education and higher professional education). Respondents who did not want to answer the question about their educational level were recorded separately.

Commonly used measures in smoking cessation research [29], which were also used in an earlier mode comparison study on smoking [30] were used in this study: (1) number of cigarettes smoked per day, (2) number of previous quit attempts, (3) time (in minutes) before smoking the first cigarette after waking, (4) attitude towards smoking (assessed by asking 'what is your overall opinion of smoking?' very positive - positive - neither positive nor negative - negative - very negative - refused - don't know), (5) intention to quit smoking (assessed by asking 'are you planning to quit smoking...' within the next month - within the next 6 months - sometime in the future, beyond 6 months - never - refused - don't know; respondents who answered "don't know" were asked what they would answer if they were forced to choose an answer), and (6) self efficacy for quitting (assessed by asking 'if you decided to give up smoking completely in the next 6 months, how sure are you that you would succeed?' not at all sure - slightly sure - moderately sure - very sure - extremely sure - refused - don't know). These questions were the same in wording across modes. Answering categories were read aloud by the telephone interviewers with the exception of the "refused" and "don't know" categories. Answering categories were visible on the screen of the web survey with the exception of the "refused" category. The "don't know" category was preceded by an extra space and was displayed in a grey font to make it less visible.

### Analyses

Because young smokers were deliberately oversampled in the web survey to address the research question of another study, all analyses were conducted with the data weighted for age. To evaluate coverage in the web sample, telephone respondents who had internet access were compared to telephone respondents without internet access. To evaluate coverage in the telephone sample, web respondents that had a fixed telephone line were compared to web respondents without a fixed line. Since coverage error was expected to affect which part of the population was contacted, respondents were compared with respect to demographic variables. This was done using chi-square tests (gender, marital status, and educational level) and t-tests (age).

To assess differences in the combination of coverage and non-response error, respondents from both samples were compared to the demographic characteristics of smoking respondents of Statistics Netherlands (CBS) from 2006-2007. The CBS statistics are the official national statistics for the Netherlands, and use the national registry as a sampling frame. The CBS uses computer assisted personal interviewing on a sample of 10,000 persons. Survey interviewing occurs evenly throughout the year. Since non-response error also was expected to affect which part of the initial sample responds to the survey, respondents were compared with respect to demographic variables. The web, telephone, and CBS samples were compared on demographic variables using chi-square tests (gender, marital status, and educational level).

To assess differences which might be attributed in large part to differences in measurement error, web and telephone respondents were compared with each other. Measurement error was expected to arise from reactions to the measurement instrument. The web and telephone samples were therefore compared on smoking related questions using chi-square tests and Mann-Whitney U-tests (attitude towards smoking, intention to quit smoking, and self efficacy for quitting) and t-tests and Levene's F-tests (number of cigarettes smoked per day, number of previous quit attempts, and time before smoking the first cigarette after waking). These analyses were employed without respondents who had no fixed telephone or who did not have access to the internet to control for coverage error differences. Furthermore, linear regression analyses were employed that tested the effects of interviewing modes on answers to smoking related questions, controlling for gender, marital status, educational level, and age. Also, interactions between interviewing mode and educational level were tested.

## Results

Both the web and telephone survey had a cooperation rate of 78.1%, which means that 78.1% of the eligible respondents who were contacted and capable of doing the interview completed the interview (see Table 1). The response rate for the telephone survey was 4.2%. This means that 4.2% of all eligible telephone respondents, including non-contacts whose eligibility was estimated, completed the interview. A response rate could not be calculated for the web survey, since the initial sample consisted of only eligible respondents who were all e-mailed. Therefore, there were no non-contacts in the web survey.

**Table 1:** Response information of the web and telephone survey with cooperation and response rates

	Web survey	Telephone survey
Eligible*	**2,331**	**592**
Interviews	1,820	404
Refusals	511	113
Non-contact	-	52
• adult smoker in household	-	3
- estimated to be eligible (97,1%)	-	3
• unknown if adult smoker in household	-	49
- estimated to be eligible (22,4%)	-	11
Other	-	23
Ineligible**	-	**11,504**
Eligibility unknown***	-	**16,467**
Unknown if housing unit	-	5,220
- estimated to be eligible (22,4%)	-	1,169
Housing unit	-	11,247
• adult smoker in household	-	7,327
- estimated to be eligible (97,1%)	-	7,115
• unknown if adult smoker in household	-	3,920
- estimated to be eligible (22,4%)	-	878
**Total attempted to contact**	**2,331**	**28,563**
Cooperation rate†	78.1%	78.1%
Response rate‡	-	4.2%

* Eligible cases included cases where an interview was completed, refusal occurred, non-contact at a residential household or where respondents were unable to complete the interview due to language difficulties or disability.

** Ineligible cases included non-residential numbers (such as business or fax lines) and telephone numbers which were invalid. Ineligible cases also included households with no adult monthly smokers of manufactured or roll-your-own cigarettes who have smoked at least 100 cigarettes in their lifetime.

*** Cases where eligibility was unknown were recorded separately.

† Cooperation rate was calculated according to the AAPOR definition COOP4 [35]. This was the number of interviews (404 for telephone) divided by the number of eligible respondents who were contacted and capable of doing the interview (404 + 113 for telephone).

‡ Response rate was calculated according to the AAPOR definition RR4 [35]. This was the number of interviews (404 for telephone) divided by the number of respondents who were eligible or estimated to be eligible (404 + 113 + 3 + 11 + 23 + 1,169 + 7,115 + 878 for telephone). Estimates of eligibility were made using the eligibility rate of the respondents from whom the eligibility was known. The eligibility rate of households with adults smokers was 97,1% and the eligibility rate of households of which it was unknown if there was an adult smoker was 22,4%. Response rate was not calculated for the web survey, since all respondents from the initial sample were contacted and eligible.

As can be seen in Table 2, 1,438 out of 1,668 (86.2%) web respondents had a fixed line telephone. Web respondents with a fixed line telephone did not differ on gender from web respondents without a fixed line telephone (p = 0.697). Web respondents with a fixed line telephone were significantly more often married (p < 0.001), had a lower educational level (p = 0.032), and were older (p < 0.001) than web respondents without a fixed line telephone.

**Table 2:** Comparison of demographics of respondents with and without fixed line telephone and internet access

	Web sample (n = 1,668)			Telephone sample (n = 404)		
	With telephone (n = 1,438)	Without telephone (n = 230)	Significance tests	With internet (n = 359)	Without internet (n = 45)	Significance tests
**Gender (%)**			χ^2 ^= 0.15			χ^2 ^= .74
Men	52.4	53.9	(df = 1)	55.8	48.8	(df = 1)
Women	47.6	46.1		44.2	51.2	
**Marital status (%)**			χ^2 ^= 39.63***			χ^2 ^= 27.93***
Not married	41.5	63.3	(df = 3)	44.0	40.0	(df = 3)
Married	50.4	28.0		49.5	37.5	
Widowed	2.2	1.4		2.5	20.0	
Divorced	6.0	7.2		4.1	2.5	
**Educational level (%)**			χ^2 ^= 8.78*			χ^2 ^= 26.29***
Low	51.9	43.0	(df = 3)	31.0	70.7	(df = 3)
Middle	34.9	37.2		50.0	24.4	
High	12.5	18.8		15.2	2.4	
No answer	0.7	1.0		3.8	2.4	
**Age (mean; SD)**	43.3; 14.7	35.3; 12.3	t = -8.53***	41.1; 13.6	57.1; 17.4	t = 5.66***

* p < 0.05

** p < 0.01

*** p < 0.001

Of the telephone respondents, 359 out of 404 (88.9%) had internet access at home. Telephone respondents with internet access did not differ on gender from telephone respondents without internet access (p = 0.389). Telephone respondents with internet access were significantly more often married (p < 0.001), had a higher educational level (p < 0.001), and were younger (p < 0.001) than telephone respondents without internet.

In Table 3, the demographics of the web and telephone respondents were compared to the demographics of the respondents of Statistics Netherlands (CBS). The CBS, web, and telephone respondents did not significantly differ on gender (CBS with web: p = 0.255, CBS with telephone: p = 0.689). The web respondents were significantly less often married (p < 0.001) and lower educated (p < 0.001) than the CBS respondents. The telephone respondents were significantly less often married (p < 0.001) and higher educated (p < 0.001) than the CBS respondents.

**Table 3:** Comparison of demographics of the Statistics Netherlands (CBS), web and telephone sample

	CBS sample (n = 4,133)	Web sample (n = 1,668)	Telephone sample (n = 404)	Significance tests	
				CBS versus web	CBS versus telephone
**Gender (%)**				χ^2 ^= 0.16	χ^2 ^= 1.30
Men	54.2	52.6	55.3	(df = 1)	(df = 1)
Women	45.8	47.4	44.7		
**Marital status (%)**				χ^2 ^= 104.68***	χ^2 ^= 32.82***
Not married	33.1	44.2	43.6	(df = 3)	(df = 3)
Married	50.2	47.7	48.3		
Widowed	5.1	2.0	4.2		
Divorced	11.7	6.1	3.9		
**Educational level (%)**				χ^2 ^= 20.14***	χ^2 ^= 95.34***
Low	46.5	50.8	35.0	(df = 3)	(df = 3)
Middle	36.7	35.1	47.4		
High	16.6	13.3	13.9		
No answer	0.3	0.8	3.7		

* p < 0.05

** p < 0.01

*** p < 0.001

Table 4 presents the comparisons of the web and telephone sample on smoking related variables. Web and telephone respondents did not differ on mean number of cigarettes smoked per day (p = 0.591), mean number of previous quit attempts (p = 0.206), and mean number of minutes before smoking the first cigarette after waking (p = 0.492). However, there were significant differences between web and telephone respondents in the variances of the number of previous quit attempts (F = 6.11, p = 0.014) and number of minutes before smoking the first cigarette after waking (F = 4.12, p = 0.043), but not in the variance of number of cigarettes smoked per day (F = 0.65, p = 0.420). In the regression analyses we found that, controlling for gender, marital status, educational level, and age, telephone and web respondents did not differ on number of cigarettes smoked per day (Beta = 0.00, p = 0.943), number of previous quit attempts (Beta = 0.04, p = 0.135), and time before smoking the first cigarette after waking (Beta = -0.03, p = 0.190).

**Table 4:** Comparison of smoking related variables in the web and telephone sample

	Web sample (n = 1,668)	Telephone sample (n = 404)	Significance tests
**Number of cigarettes smoked per day (mean; SD)**	15.3; 8.1	15.0; 8.5	t = -0.54
**Number of previous quit attempts (mean: SD)**	2.0; 2.2	2.2; 2.5	t = 1.27
**Time (in minutes) before smoking the first cigarette (mean; SD)**	93.1; 189.2	86.5; 154.9	t = -0.69
**Attitude towards smoking (%)**			χ^2 ^= 32.12***
Very positive	1.5	0.8	(df = 6)
Positive	12.7	9.2	
Neither positive nor negative	64.3	60.0	
Negative	17.6	23.3	
Very negative	2.4	6.7	
Refused	0.0	0.0	
Don't know	1.6	0.0	
**Intention to quit smoking (%)**			χ^2 ^= 51.25***
Within the next month	5.6	7.8	(df = 5)
Within the next 6 months	19.3	20.0	
Sometime in the future, beyond 6 months	56.6	44.7	
Never	14.3	26.7	
Refused	0.0	0.3	
Don't know	4.2	0.6	
**Self efficacy for quitting (%)**			χ^2 ^= 29.38***
Not at all sure	35.4	30.6	(df = 6)
Slightly sure	35.0	29.5	
Moderately sure	16.5	22.3	
Very sure	6.0	5.6	
Extremely sure	4.5	9.7	
Refused	0.0	0.3	
Don't know	2.7	1.9	

* p < 0.05

** p < 0.01

*** p < 0.001

As can be seen in Table 4, web respondents used the "don't know" options more often than telephone respondents on the categorical variables. Telephone respondents were more negative about smoking (p < 0.001), and had less intention to quit smoking (p < 0.001), and more self efficacy for quitting, than web respondents (p < 0.001). When the "refused" and "don't know" answers were recoded to missing values, the central tendency of attitude towards smoking (U = 245,110; p < 0.001), intention to quit smoking (U = 255,932; p = 0.029), and self efficacy for quitting (U = 248,179; p = 0.004) differed significantly between interviewing modes. In the regression analyses we found that, controlling for gender, marital status, educational level, and age, telephone respondents had significantly more negative attitudes towards smoking (Beta = -0.09, p < 0.001), less intention to quit smoking (Beta = -0.07, p = 0.002), and more self efficacy for quitting (Beta = 0.06, p = 0.008) than web respondents.

When controlling for gender, marital status, educational level, and age, interactions of mode of interviewing with educational level were tested with the smoking related variables as outcomes. There was a significant interaction effect found of mode of interviewing with educational level on self efficacy for quitting (Beta = -0.15, p = 0.003). Higher educated respondents had more self efficacy for quitting in the web sample (Beta = 0.17, p < 0.001), but not in the telephone sample (Beta = 0.05, p = 0.314).

## Discussion

The aim of this study was to compare the ITC Netherlands web and telephone surveys on demographic and smoking related variables to assess differences in data quality. Data quality differences were present, but they were small and not consistently favourable for either web or telephone interviewing. Cooperation rates were high for both the web and telephone survey (78%), which can be explained by the use of a well respected market research company and the use of reimbursements.

In our study we found relatively large coverage error differences. For example, 31% of respondents with internet access had a low educational level, while 71% of respondents without internet access had a low educational level. However, in absolute terms, coverage error differences are not very large in the Netherlands, since there are not many people without internet access (14% of the population) or without a fixed line telephone (9% of the population) [17]. As internet access keeps increasing and fixed line telephone access keeps decreasing, as was the case in the last several years [17,31,32], the degree of coverage error in web surveys is expected to decrease while the degree of coverage error in telephone surveys is expected to increase. Our web and telephone samples both showed coverage error differences with respect to marital status, educational level, and age, but not with respect to gender. Differences between telephone respondents with and without internet access were somewhat larger than differences between web respondents with and without fixed line telephone.

Non-response combined with coverage error differences were found for both the web and telephone survey with respect to marital status and educational level, but not with respect to gender. The largest difference was found between the telephone survey and the Dutch population of smokers with respect to educational level. The telephone survey contained 12% less lower educated respondents than the Dutch population of smokers. In order to be able to generalise the findings of these surveys to the Dutch population of smokers, weighting the data to this population seems crucial.

Measurement error differences were found on three of the six smoking related variables. Although these differences were significant, they were only minor: the mean differences in the use of answering categories between the web and telephone survey was 4%. Web respondents used the "don't know" options more often than telephone respondents, as is in accordance with what was found in other studies [2,11,12]. This occurs primarily because web respondents see the "don't know" option on the screen whereas telephone interviewers do not read it aloud. Another possibility is that web respondents feel less pressure to give an answer when they do not know the answer to the question [2]. Furthermore, telephone respondents were more negative about smoking and had more self efficacy for quitting than web respondents. These differences might be caused by more socially desirable responding with telephone than with web interviewing [2,12,16]. Also, telephone respondents answered more often that they were not planning to quit smoking, while web respondents answered more often that they were planning to quit smoking sometime in the future but not within 6 months. This difference might be caused by more balanced answering with web interviewing, due to less perceived time pressure [2]. According to another study with ITC data from the United States, Canada, the United Kingdom, and Australia, more highly educated smokers have more self efficacy for quitting [25]. This was also found with the ITC Netherlands web survey, but not with the telephone survey, suggesting that the web survey results may be more valid than the telephone survey results.

In this study, we also found some potential disadvantages of web interviewing. Smokers with internet access had a higher educational level than smokers without internet access. Since we used a quota sample with among others quotas for educational level for the web survey, this difference did not result in a higher educated web sample. Therefore, we recommend using quotas for educational level when using web interviewing. Another potential disadvantage of web interviewing is the relatively high number of "don't know" answers. In our web survey, the "don't know" category was visible on screen in a grey font and preceded by an extra space to prevent greater use of the "don't know" option. Answering "don't know" was still relatively high with the question about intention to quit smoking among web respondents (4%). This can be solved by recoding "don't know" answers as "no intention to quit smoking", which is already common practice in studies with one interviewing mode [29,33] and can reduce the differences between a web and telephone sample in mixed mode studies.

Of course, factors other than survey error play a role when choosing an interviewing mode for a particular study. Web surveys are less costly than telephone surveys, and allow for lengthier questionnaires, a shorter fieldwork period, and the inclusion of visual stimuli [1-3]. Budget constraints can be a powerful motivator to choose web interviewing, since the differences in costs are huge. In our study, the costs were €15 (US$22) for fieldwork costs and reimbursements per web respondent and €62 (US$90) per telephone respondent. Some studies suggest that this difference will increase in time, because telephone surveys become increasingly more expensive [1,34].

Although our study shows that using a mixed mode approach can threaten comparability of results, it can also have advantages. A mixed mode approach can reduce coverage error, when people without access to one mode have access to another mode [1,3,6]. Also, using a mixed mode approach in multi-country studies with one mode within each country but different modes across countries can reduce coverage error when one country has high internet access and low telephone access and another country has high telephone access and low internet access [1,3,6]. Furthermore, non-response error can be reduced by using a mixed mode approach in which respondents can choose their interviewing mode [3,6]. Finally, measurement error can be reduced by using a self-administered interviewing mode (for example web interviewing) for answering the sensitive questions that are part of an interviewer administered survey (for example telephone interviewing) [3,6].

### Limitations

A limitation of our study was that we could not compare the age distributions of our surveys with the Dutch population of smokers. This was due to a deliberate overrepresentation of young smokers in the web sample, for the purpose of another study. This was unfortunate, since age is an important factor in smoking behaviour [23,24]. Fortunately, we could compare the three samples on education, which is perhaps an even more important factor in smoking behaviour. Since the web and telephone sample both contained more respondents who were not married than the Dutch population of smokers, it is expected that they also contained more younger respondents.

Another limitation was that we used internet access at home as an indicator of the possibility to participate in web surveys. However, internet access at school, work, and at other public places can also give the possibility to participate in web surveys. Our study may therefore have overestimated the problem of coverage error in web interviews.

The Netherlands is a country where 86% of the population has internet access, where 88% of the internet users have a broadband connection [17], and where web surveying is common practice. Therefore, the results from this study may not be generalisable to countries with lower levels of internet access and less experience with web surveying. However, with increasing internet access and the increasing use of web surveys worldwide, the results from this study may well apply to other countries in the near future.

## Conclusion

Web interviewing can be regarded as a good alternative to telephone interviewing in smoking cessation research. This conclusion is based on the facts that both our web and telephone surveys contained coverage and non-response error differences, which were not consistently favourable for either web or telephone interviewing. Differences between the web and telephone surveys on smoking related variables were small. There were indications of more socially desirable responding with telephone interviewing, suggesting that web surveys may even obtain better data quality than telephone surveys.

## List of abbreviations

ITC Project: International Tobacco Control Policy Evaluation Project; DCSSH: Dutch Continuous Survey of Smoking Habits; CBS: Statistics Netherlands.

## Competing interests

The authors declare that they have no competing interests.

## Authors' contributions

GN conducted the statistical analyses and wrote the manuscript. MW, MT, GF, BvdP, and HdV advised on the design of the study and the writing of the manuscript. All authors read and approved the final manuscript.

## Pre-publication history

The pre-publication history for this paper can be accessed here:

http://www.biomedcentral.com/1471-2458/10/351/prepub
